# Supplementary material for: Combination treatment to improve mucociliary transport of Pseudomonas aeruginosa biofilms
Source: PLoS One. 2024 Feb 23;19(2):e0294120. doi: 10.1371/journal.pone.0294120 (PMC10890754; doi:10.1371/journal.pone.0294120)
Supplement: S1 File — (DOCX) [file pone.0294120.s001.docx]

**Supplemental Information**

Supplemental Table 1. Post hoc filtering of mucociliary transport data for treated PAO1 biofilms.

| # Beads | PAO1 | Tob | TCEP 10 | TCEP+Tob | DNase | DNase+Tob | HA6 | HA+Tob | NP40 | NP+Tob |
| --- | --- | --- | --- | --- | --- | --- | --- | --- | --- | --- |
| Original | 268 | 675 | 237 | 473 | 391 | 174 | 1144 | 989 | 141 | 188 |
| Post filter | 253 | 239 | 231 | 398 | 371 | 165 | 416 | 734 | 116 | 160 |
| Removed: | 15 | 436 | 6 | 75 | 20 | 9 | 728 | 255 | 25 | 28 |
| % Retained | 94% | 35% | 97% | 84% | 95% | 95% | 36% | 74% | 82% | 85% |


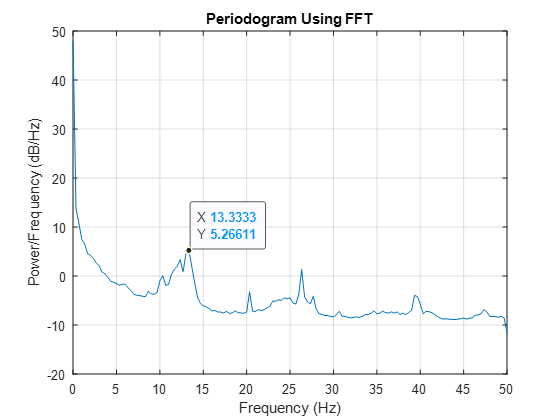


Supplemental Figure 1. Representative periodogram of cilia beat frequency quantification.


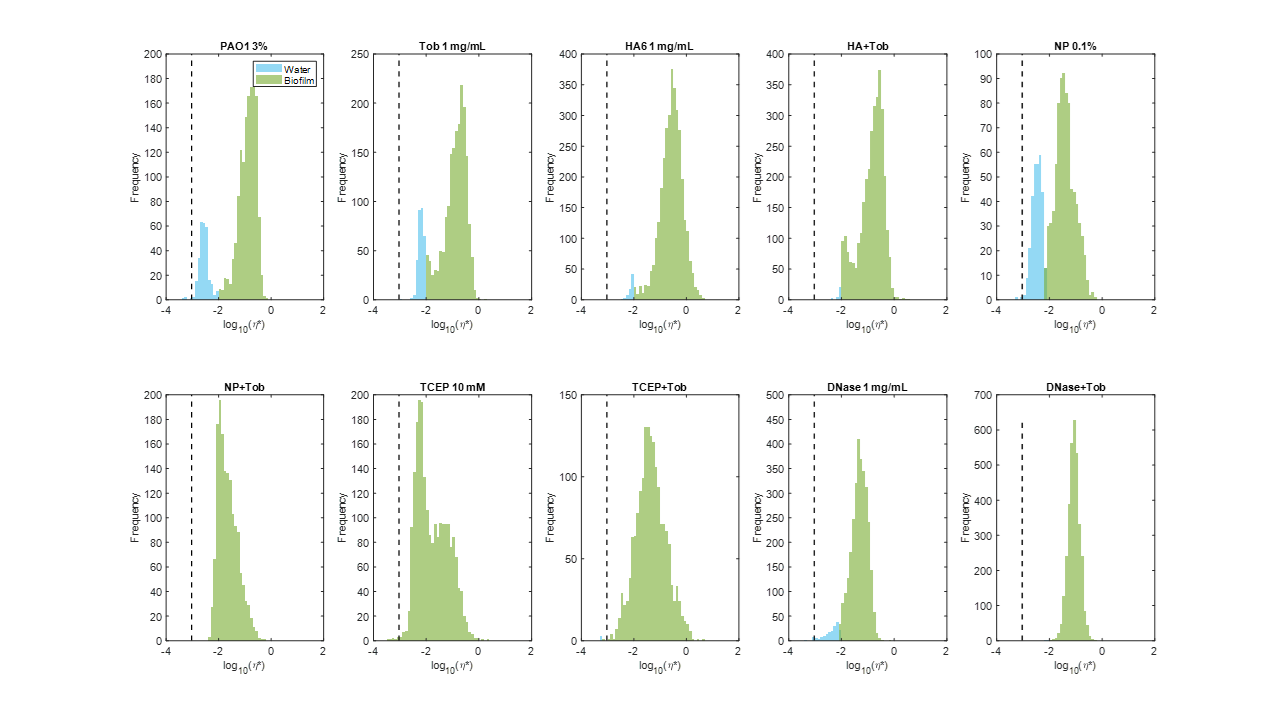
Supplemental Figure 2. Complex viscosity distribution of treated PAO1 biofilms. Gaussian mixture modeling was used to separate the watery component (blue) from the more solid-like biofilm component (green).


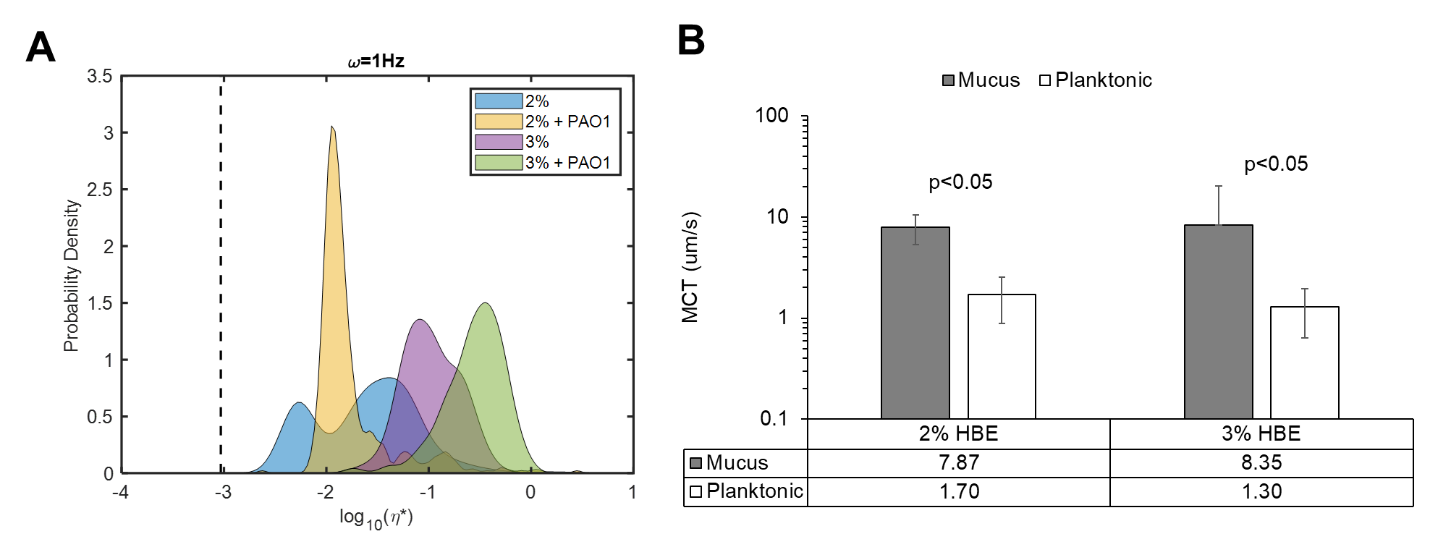


Supplemental Figure 3. Effect of planktonic bacteria on A) mucus rheology and B) transport. Statistical significance was determined using single factor ANOVA with post hoc Tukey Kramer analysis.


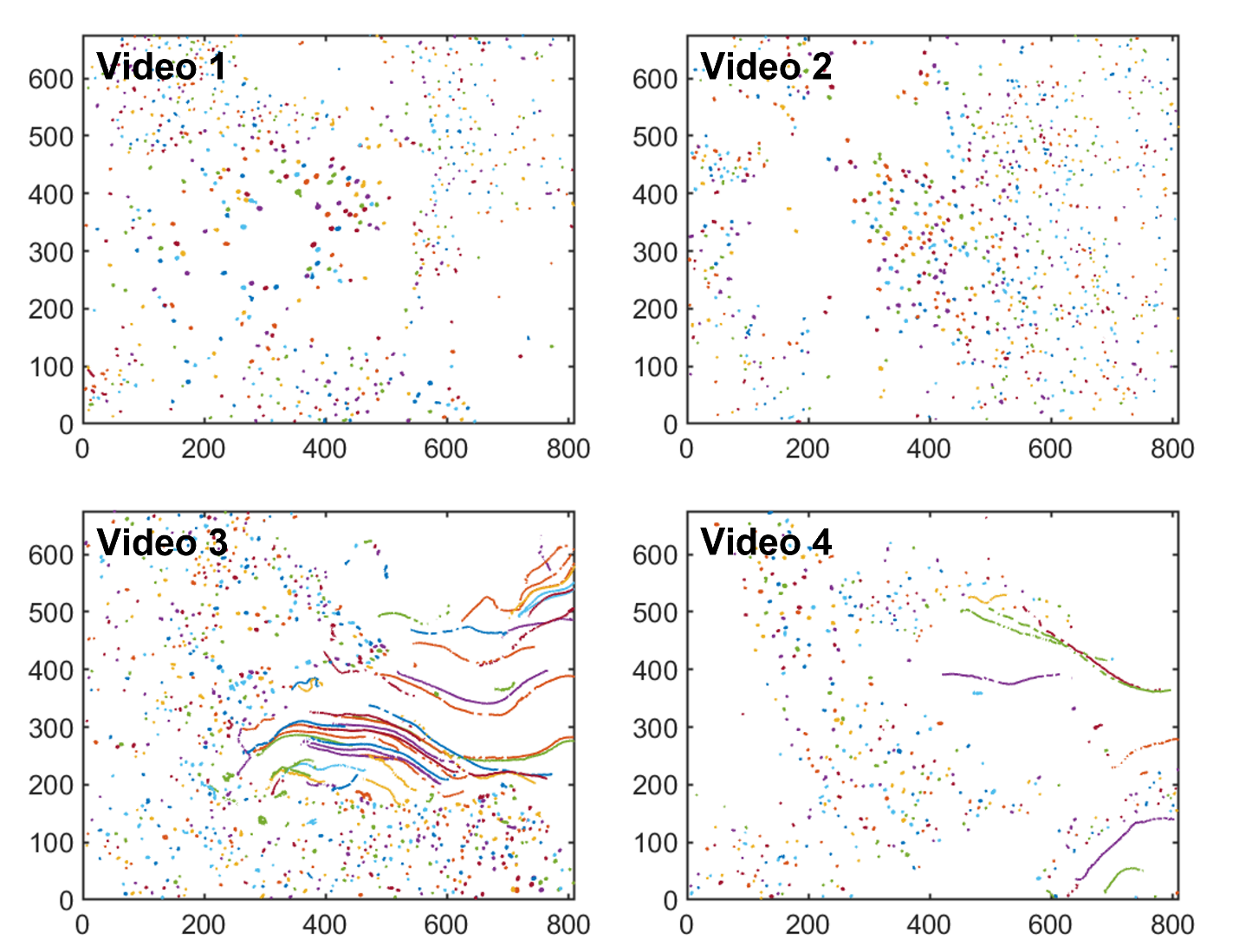


Supplemental Figure 4. Representative images of biofilm mucociliary transport after treatment with 10 mM TCEP and 1 mg/mL Tob. Each video is taken from a different region of the biofilm within the culture.


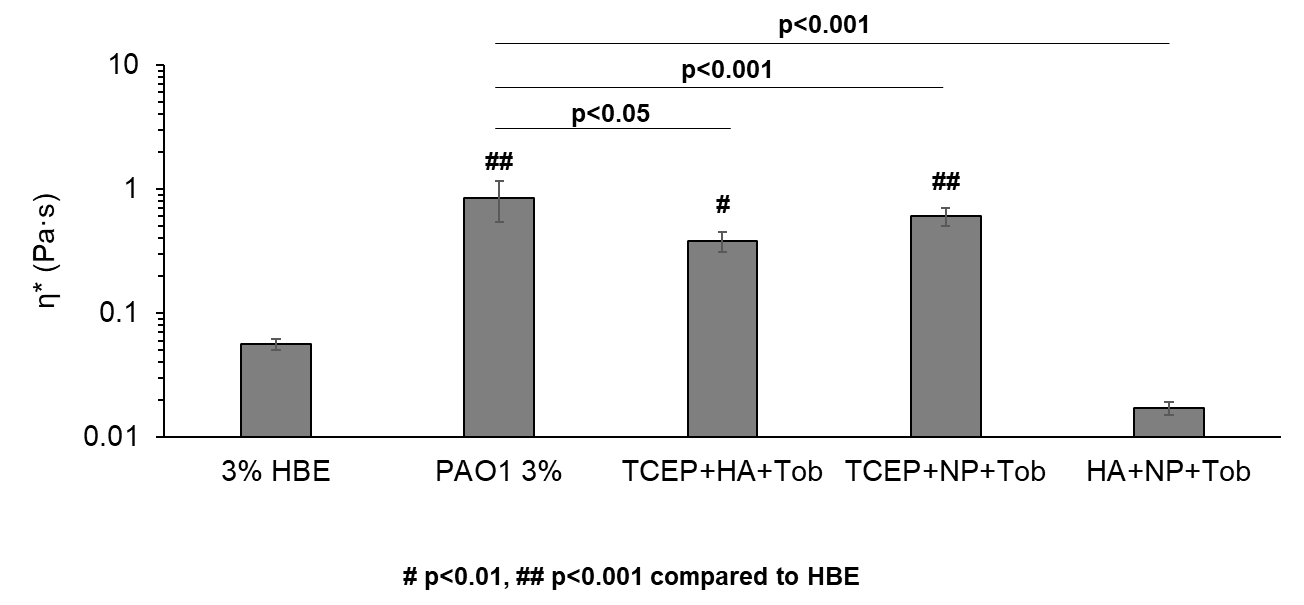


Supplemental Figure 5. Macrorheology of triple combination treated biofilms. Data is representative of the average ± standard deviation of three separately treated and evaluated samples. Statistical significance was determined with single factor ANOVA with post hoc Tukey analysis.


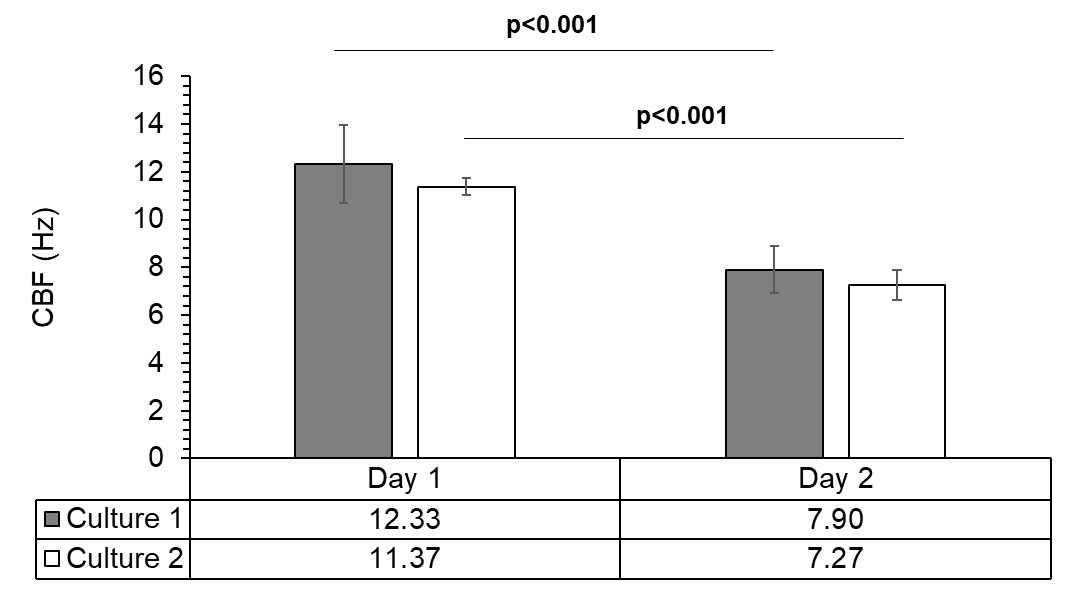
 Supplemental Figure 6. Cilia beat frequency of two racetrack cultures as a function of time post infection with a PAO1 biofilms. Data is representative of the average ± standard deviation of six separate videos for each culture. Statistical significance was determined with single factor ANOVA with post hoc Tukey analysis.
