# Supplementary material for: Combination treatment to improve mucociliary transport of Pseudomonas aeruginosa biofilms
Source: PLoS One. 2024 Feb 23;19(2):e0294120. doi: 10.1371/journal.pone.0294120 (PMC10890754; doi:10.1371/journal.pone.0294120)
Supplement: Attachment — Submitted filename: MCT response to reviewers.docx [file pone.0294120.s002.docx]

We thank the reviewers for their kind and constructive review of our manuscript. We have addressed each concern with tracked changes in the submitted document. Below are our point-by-point replies:

Reviewer 1:

1. NP40 is nonyl phenoxypolyethoxylethanol. We have updated the manuscript to include this full name.
2. NP40 was used at 0.1% which is below the critical micelle concentration. We wanted to avoid any complications from micelle formations in the rheological impact of treatment. Additionally, the 0.1% dose was the lowest dose necessary to induce significant decreases in macroscopic and microscopic complex viscosity values in both 2% and 4% mucus. These data are included in a different manuscript submission specific to mucus rheology. We have referenced the previous study that included the dosages used for the other components.
3. The removal of the stuck beads is critical for an accurate quantification of MCT rates. A large portion of the beads have effectively transport rates of zero, which decreases the ensemble rate greatly. The number of stuck beads across videos ranges from 0-25, depending on the properties of the transporting sample. More heterogeneous and viscous samples are associated with more stuck beads, such as untreated biofilms and mucus + planktonic bacteria. The treated biofilms that have a compromised matrix, particularly those treated with the triple combinations, have far fewer stuck beads. For transparency, we have included the number of stuck beads removed from each sample into a table in the supporting information.
4. Each video is acquired in a different region of the culture. We have updated the caption to specify this.

Reviewer 2:

1. Transport in diseased HBE cells can be especially challenging, particularly with decreased MCT rates in the diseased cultures. Also, with the decreasing availability of CF lungs, CF cells are being used for more precious experiments. We are currently investigating the use of conditionally reprogrammed cells (CRC) to expand CF cells to compare mucus biophysical properties. When these studies are complete, we will be able to utilize CF HBE mucus and cells for transport experiments. For the purpose of this study, HBE mucus was prepared at 3% solids to represent mild pulmonary disease. We believe that the present work lays the foundation necessary for more complex studies with diseased HBE cells, like CF HBE CRC cultures, that can then benefit from a dose-response study with the triple combination treatments screened in this study.
2. Treatments that maximized biofilm disruption (increase or decrease in biofilm complex viscosity) and reductions in bacterial viability were combined with tobramycin for the triple combination treatment. DNase was excluded from triple combination treatments due to minimal impact on biofilm rheology in high mucus concentrations, which we have published on previously. TCEP, NP40, and HA have demonstrated more promising biofilm disruption capabilities in this study and our previous work. This study laid the groundwork necessary to evaluate a single treatment (TCEP/NP40/HA) in an infected murine model, which is upcoming.
3. Throughout the document, PAO1 3% is used to indicate biofilms grown in 3% mucus while 3% HBE + PAO1 is used to indicate mucus with planktonic bacteria. The caption includes this information as well. We have also included a clarifying statement into the text to highlight the nomenclature and remove any confusion.
4. The individual agents do not significantly decrease bacterial viability, and ultimately, the goal is to optimize reductions in biofilm complex viscosity and viability. Thus, we must ensure that there are no antagonistic interactions between the biofilm disruption agent (e.g., TCEP) and the antibiotic.
5. We have previously published on the non-Gaussian parameter (κ) for PAO1 biofilms grown in mucus. Greater κ values indicate greater heterogeneity of the biofilm biophysical properties, which may be used as a measure of biofilm disruption. The combinations that induced the greatest changes in κ values were TCEP, HA, and NP40, each with tobramycin. The PTMR data for each of these conditions is included in Supplemental Figure 2 where we qualitatively observe changes in biofilm biophysical behavior (i.e., complex viscosity distribution). These values suggest that combinations of these agents may result in superior biofilm disruption that may be associated with increased MCT rates. Indeed, the triple combinations of these agents significantly increased MCT rates. We have added a statement to this effect into the discussion section.
6. The CFU data for the combinations are included in Figure 4, but treatment with the single agent had little effect on viability. We hypothesize that biofilm disruption with the single agent (e.g., TCEP, HA) was sufficient to increase MCT rates but not eradicate bacteria. However, the addition of tobramycin resulted in cell death that contributed to slightly increased complex viscosity values (4C) and decreased MCT rates (Fig 5). We have included a statement to this effect in the results section.
7. We have a reproducible and robust biofilm growth model in HBE mucus with PAO1 for in vitro studies. The benefit to PAO1 for these experiments is its broad availability to other labs as well. The goal of this work was to screen and evaluate combination treatments in order to select an optimal in vitro candidate for in vivo studies. Because the in vitro susceptibility of even clinical isolates is often different from in vivo behavior, we wanted to optimize biophysical changes with PAO1 before moving to an isolate that’s been optimized for an infected murine model. These studies are ongoing.
